# Tibialis anterior pennation angle at ICU admission and 60-day mortality in critically ill patients: a prospective observational study

**DOI:** 10.3389/fmed.2026.1815137

**Published:** 2026-04-22

**Authors:** Yu Zhou, Lulu Wan, Xiaopeng Li, Guangzhen Li, Hailun Peng, Qiang Chen, Yazhen Zeng, Jingyi Huang, Jianfeng Wu, Ronglin Chen, Fei Pei, Bin Gu, Xiangdong Guan

**Affiliations:** 1 Department of Critical Care Medicine, The First Affiliated Hospital, Sun Yat-sen University, Guangzhou, Guangdong, China; 2 Guangdong Clinical Research Center for Critical Care Medicine, Guangzhou, Guangdong, China; 3 Department of Critical Care Medicine, Longgang Central Hospital, Shenzhen, Guangdong, China; 4 Department of Rehabilitation Medicine, The First Affiliated Hospital, Sun Yat-sen University, Guangzhou, Guangdong, China; 5 Sino-French Engineer School, Beihang University, Beijing, China

**Keywords:** 60-day mortality prediction, muscle ultrasound indices, nomogram, pennation angle, tibialis anterior

## Abstract

**Background:**

Skeletal muscle is an important organ strongly associated with prognosis in critically ill patients. 60-day mortality represents a key endpoint for evaluating the transitional phase from ICU survival to functional recovery, yet the association between ultrasound-derive muscle indices at ICU admission and 60-day mortality remains unclear.

**Methods:**

This dual-center, prospective observational study was performed from January to December 2024. Four ultrasound indices of muscle quantity [thickness and cross-sectional area of the rectus femoris (RF-TH and RF-CSA), thickness of the vastus intermedius (VI-TH), thickness of the quadriceps femoris (QF-TH)], and one index of quality (tibialis anterior pennation angle, TA-PA) were assessed within 24 h of ICU admission. The primary endpoint was 60-day all-cause mortality. To identify predictive factors, multivariable logistic regression analysis was employed. Additionally, a predictive nomogram model was developed.

**Results:**

A total of 247 critically ill adult patients were included, with a median age of 61.0 years (51.0–72.0), including 171 males (69.20%). During 60-day follow-up, 53 patients (21.50%) died. Compared with the survivors, the non-survivors exhibited significantly lower RF-TH (0.54 [0.43–0.70] vs. 0.64 [0.48–0.85]cm; *P* = 0.022), VI-TH (0.64 [0.47–0.86] vs. 0.77 [0.64–1.04]cm; *P* = 0.002), QF-TH (1.32 [0.96–1.63] vs. 1.48 [1.24–1.97]cm; *P* =0.005) and TA-PA (7.08[5.63–8.48] vs. 7.94[6.62–9.32]; *P* =0.002). Among them, TA-PA was identified as an independent predictor of 60-day mortality (β = −0.216, *P* = 0.035). TA-PA, in conjunction with APACHE II score and prealbumin level, constructed a predictive nomogram, with a consistency index (C-index) of 0.785 (95%CI: 0.728–0.834). Calibration assessed by Spiegelhalter *Z*-test showed *P* > 0.05 indicating adequate calibration for the predicted and observed models. Decision curve analysis (DCA) confirmed that the nomogram prediction model had good clinical benefits. Internal validation demonstrated stable model performance, with a concordance statistic (C-index) of 0.785. Compared to the ROC curve based solely on APACHE II score, the nomogram exhibited significantly higher area under the curve (AUC) (0.785, 95%CI: 0.728–0.834 vs. 0.726, 95%CI: 0.666–0.781, *P* = 0.023).

**Conclusions:**

Lower TA-PA at ICU admission is an independent predictor of 60-day mortality. Incorporating this ultrasound-derived muscle quality index with APACHE II score and prealbumin level may improve early risk stratification in critically ill patients.

## 1 Introduction

Skeletal muscle constitutes for approximately 40% of body mass and plays a crucial role in facilitating physical movement, maintaining posture, and performing vital functions such as mastication, swallowing, and respiration (1). Additionally, it is the primary site for glucose uptake and storage and serves as an amino acid reservoir, supporting either protein synthesis or energy production. During periods of nutritional deprivation, sufficient muscle can help maintain the protein content of vital tissues and organs and stabilize plasma glucose concentrations by providing the necessary amino acids (2). Previous studies have demonstrated that skeletal muscle status is closely associated with clinical prognosis across various diseases (3–5).

Muscle quantity and quality collectively determine skeletal muscle status. Whole-body muscle quantity is commonly evaluated using bioelectrical impedance analysis (BIA) and dual-energy X-ray absorptiometry (DXA), while regional muscle quantity is typically assessed through computed tomography (CT), magnetic resonance imaging (MRI), or ultrasound. Common parameters for these assessments include muscle cross-sectional area (CSA) and thickness. Muscle quality is defined as both micro and macro structures and compositions of muscles, which is closely related with muscle strength (6). It is often evaluated using CT or ultrasound, with primary indicators including skeletal muscle density (SMD), myosteatosis, fascicle length, pennation angle, echo intensity (EI) and shear-wave elastography (SWE) (7). In critically ill patients, lower CT-derived skeletal muscle quantity or quality at intensive care unit (ICU) admission, such as reduced pectoralis muscle area (8), reduced erector spinae muscle (9), lower SMD (10), has been associated with adverse clinical outcomes, including prolonged mechanical ventilation duration and increased mortality rates. While CT is expensive and inconvenient for ICU patients, ultrasound is emerging as a potentially powerful tool for skeletal muscle assessment due to its advantages of being non-invasive, painless, cost-effective, and portable.

The most studied ultrasound-derived muscle quantity indices include the thickness of the quadriceps femoris (QF-TH), as well as the thickness and cross-sectional area of the rectus femoris (RF-TH and RF-CSA). These researches mainly focused on the relationship between muscle indices and the response to nutritional therapy or prognosis, such as mechanical ventilation duration, hospital stay, and ICU mortality (11–14). Few studies have specifically investigated muscle quality indices in critically ill patients. Among patients undergoing ventilator weaning, the weaning failure group had significantly higher EI of respiratory muscle, suggesting adipose tissue infiltration within the muscular structure, which consequently leads to decreased muscle strength (15). In COVID-19 patients, survivors with reduced muscle strength had higher media gastrocnemius SWE compared to those with normal muscle strength. SWE is an indicator of muscle stiffness, and is inversely correlated with muscle strength (16).

Current research on mortality in critically ill patients primarily focuses on quantitative ultrasound muscle indices and short-term mortality outcomes (17, 18). The 60-day mortality rate is important for evaluating the transitional phase from ICU survival to functional recovery, enabling identification of patients who survive the acute illness but remain at heightened risk of death within this window due to impaired rehabilitation (19). In addition to the muscle loss observed during the acute phase, which is prognostically significant, muscle serves as a crucial reserve organ, and their baseline condition is closely linked to prognosis (20). Therefore, this study was conducted to evaluate the association between the baseline ultrasound-derived indices of muscle quantity and quality and the 60-day mortality, aiming to provide novel insights to support early risk stratification of mortality in critically ill patients.

## 2 Materials and methods

### 2.1 Study design

This dual-center, prospective, observational study was performed in ICU of the First Affiliated Hospital of Sun Yat-Sen University and the Longgang Central Hospital of Shenzhen from January to December in 2024. The primary observational endpoint was 60-day all-cause mortality. The Eligible patients underwent a muscle ultrasound examination within 24 h of admission to ICU. Demographic data, laboratory test results obtained within this timeframe were systematically recorded. The study protocol was approved by the Ethics Committee of the First Affiliated Hospital of Sun Yat-sen University ([2024]254-1) and the Longgang Central Hospital of Shenzhen (2024ECPJ077).

### 2.2 Sample size calculation

We calculated the sample size on the basis of the events per variable (EPV) metric, a widely accepted method in statistical analysis (21). Drawing upon historical data from our center's database (22) as well as findings from prior literature (23, 24), the 60-day mortality rate for ICU patients is observed to range around between 19.7 and 34.6%. Consequently, we have established the mortality rate at 23%. Given our intention to include four predictor variables and set the EPV to 10, we calculated the required sample size via the following formula:


$$\begin{array}{l} Sample\;Size=\frac{Number\;of\;Variable\;\times \;EPV\;}{Incidence\;Rate}=\frac{\;4\;\times \;10}{0.23}=174 \end{array}$$

Moreover, taking into account an estimated 10% loss to follow-up rate, the calculated sample size amounts to 194.

### 2.3 Study population

Each patient admitted to ICU was screened, and inclusion criteria were: 1) Age ≥18 years; 2) Obtain informed consent from the patient or authorized relatives. Exclusion criteria: 1) Fracture or defect of lower limb; 2) Have primary motor dysfunction or definite neuromuscular disease; 3) Skin trauma or infection at measurement sites; 4) burnt area ≥50%TBSA); 5) Lower extremity artery embolism; 6) Lower extremity venous thrombosis; 7) Uncontrollable muscle fibrillation; 8) Unable to maintain the measuring position; 9) Pregnancy; 10) Postoperative resuscitation.

### 2.4 Data collection

Demographic characteristics, including age, sex, and body mass index (BMI), were systematically collected. Additionally, the Acute Physiology and Chronic Health Evaluation II (APACHE II), the Sequential Organ Failure Assessment (SOFA), and the Nutritional Risk Screening 2002 (NRS 2002) within 24 h following ICU admission were documented. Diagnoses and comorbid conditions documented at the time of ICU admission were recorded. Laboratory tests conducted within 24 h of ICU admission included measurements of hepatic and renal function markers, nutritional parameters, and complete blood counts. Furthermore, data on the duration of mechanical ventilation, length of stay in the ICU, and total hospital stay were digitally recorded and extracted from the hospital database. The 60-day mortality outcome was determined through follow-up phone interviews conducted by an independent investigator.

### 2.5 Muscle ultrasonography and measurement

In this study, four muscle quantity indices [thickness of the rectus femoris (RF-TH), vastus intermedius (VI-TH) and quadriceps femoris (QF-TH), cross-sectional area of the rectus femoris (RF-CSA), thickness of the tibialis anterior (TA-TH)], and one quality index (tibialis anterior pennation angle, TA-PA), were measured for further analysis. A 3–13 MHz linear probe from the M9 ultrasound machine (Shenzhen Mindray Bio-Medical Electronics Co., Ltd.) was employed at two research centers. Participants were positioned supine with the knee joint passively extended and the ankle joint maintained in an anatomically neutral position. The head of the bed was elevated to 30°, while the foot of the bed remained horizontal. Measurements for all participants were consistently taken from the right lower limb. Thigh circumference (TC) was determined by gently encircling the thigh with a measuring tape at the midpoint between the inguinal crease and the proximal border of the patella (25). Cross-sectional images of the thigh muscles were captured at two-thirds of the distance from the anterior superior iliac spine to the upper border of the patella, perpendicular to the thigh's long axis (26). The probe was applied with an adequate amount of gel and minimal pressure to ensure complete image acquisition. The operator adjusted the probe's position and scanning depth until the femur was visible, allowing for the clear and complete delineation of the rectus femoris cross-sectional area (27). The measurements of the RF-TH, RF-CSA, VI-TH, and QF-TH are depicted in Supplementary Figure S1. For the PA assessment, the ultrasound probe was positioned at the upper one-third point from the lateral space of the knee joint to the lateral malleolus, aligned parallel to the long axis of the tibia. The probe was gently rotated until a clear view of the parallel intramuscular fiber bundle and deep aponeurosis was achieved. The angle formed between these structures was identified as the TA-PA (28), as shown in Supplementary Figure S1. Ultrasound scans were collected as soon as possible after ICU admission and at most within the first 24 h, in order to minimize the effects of muscle loss related to the intensive care environment and to ensure that the measurements reflected the patients' baseline muscle status as closely as possible. For each region, two images were acquired employing the integrated measurement scale of the ultrasound device, and the mean value was subsequently utilized for further analysis. Data collection was independently performed by three researchers. Prior to patient enrollment, all researchers involved in ultrasound measurements underwent standardized training. After testing on 10 patients, the Intra-class Correlation Coefficient (ICC) analysis was performed. Both intra-observer and inter-observer consistency exceeded 0.9, as detailed in Supplementary Table S1.

### 2.6 Statistical analysis

Baseline characteristics were summarized as means with standard deviations (SDs) for continuous variables with normal distributions, medians with interquartile ranges (IQRs) for continuous variables with non-normally distributions, and frequencies with percentages for categorical variables. Normality of continuous variables was assessed using the Shapiro-Wilk test. Group comparisons for normally distributed continuous variables were conducted using the independent samples *t*-test. For non-normally distributed continuous variables, the Mann-Whitney *U*-test was employed. Categorical variables were compared between groups using chi-square test, with Fisher's exact test applied where expected cell counts were less than five. A two-way mixed-effects model was employed to perform ICC analysis, focusing on the consistency of average measures. For each variable, the ICC estimate along with its 95% confidence interval was calculated. Univariable and multivariable logistic regression was used to evaluate associations between candidate predictors and 60-day mortality. Variables exhibiting a variance inflation factor (VIF) above 10 were excluded from the multivariable model. Missing data were shown in Supplementary Table S2, and were imputed using multiple imputation by chained equations (MICE) with predictive mean matching. Given the maximum miss rate of 8.1% for prealbumin, five imputed datasets were generated, which was considered adequate according to methodological guidelines (29). Each imputed dataset was analyzed separately using multivariable logistic regression, and the results were pooled using Rubin's rules to obtain combined estimates. Subsequently, a nomogram was developed to predict 60-day mortality utilizing a multivariable model consisting of optimal predictors. The discrimination of the model was assessed using the concordance index (C-index), which corresponds to the area under the receiver operating characteristic (ROC) curve. Calibration was evaluated by plotting observed vs. predicted probabilities using a calibration curve, and statistically using the Spiegelhalter *Z*-test. Decision curve analysis (DCA) was performed to determine the net benefit threshold of prediction. Internal validation was performed using bootstrapping with 1,000 resamples to calculate optimism in the C-index and calibration metrics, and an optimism-corrected C-index was reported. Receiver operating characteristic (ROC) curve of APACHE II and integrated model were compared using Delong test. Schematic diagram was constructed using BioRender (https://www.biorender.com/). SPSS software (version 25.0, IBM Corp., New York, USA), and R studio software (version 4.4.1, R Foundation for Statistical Computing, Vienna, Austria) were used for data analysis and mapping. All statistical tests were double-tailed, and *P* < 0.05 was statistically significant.

## 3 Results

In this study, 712 patients were screened across two medical centers from January to December 2024. In accordance with eligibility criteria, 251 patients underwent muscle ultrasound examinations within 24 h of ICU admission in the study period. Of these, four patients were lost to follow-up within 60 days, and 247 patients were included in the final analysis (Supplementary Figure S2).

### 3.1 Patient characteristics

Baseline demographics are shown in Table 1. The median age was 61.0 years (IQR: 51.0–72.0), the majority were male (171 [69.2%]), the median SOFA score was 8.0 (IQR: 5.0–10.0), and the median APACHEII score was 16.0 (IQR: 12.0–21.0), and the median NRS2002 score was 4.0 (IQR: 3.0–5.0). During the 60-day follow-up, the all-cause mortality rate was 21.5% (53 deaths among 247 patients). Compared to survivor group, non-survivor group had higher SOFA scores (11.0 [6.5–14.0] vs. 7.0 [5.0–10.0]; *P* < 0.001), higher APACHE II scores (21.0 [15.5–26.0] vs. 15.0 [11.0–19.0]; *P* < 0.001), and higher NRS2002 scores (5.0 [4.0–6.0]) vs. 4.0 [3.0–5.0]; *P* = 0.001) at ICU admission. Besides, non-survivor group exhibited a higher incidence of sepsis diagnosis (62.3 vs. 40.7%; *P* = 0.005), lower level of platelet (107.0 [61.5–184.0] vs. 163.5 [106.5–219.3] × 10^9^/L; *P* = 0.001), lower level of prealbumin (78.0 [55.5–119.5] vs. 107.0 [81.5–148.3] mg/L; *P* < 0.001).

**Table 1:** Comparison of characteristics between 60-day survivors and non-survivors at ICU admission.

Items	Total (*n* = 247)	Survivors (*n* = 194)	Non-survivors (*n* = 53)	*P*
Age (years)	61.00 (51.00–72.00)	61.0 (51.00–71.25)	61.00 (50.50–72.00)	0.682
Sex (male, *n* (%))	171 (69.2%)	132 (68.0%)	39 (73.6%)	0.504
BMI (kg/m^2^)	22.57 ± 3.58	22.63 ± 3.61	22.35 ± 3.52	0.616
SOFA	8.0 (5.0–11.0)	7.0 (5.0–10.0)	11.0 (6.5–14.0)	<0.001
APACHE II	16.0 (12.0–21.0)	15.0 (11.0–19.0)	21.0 (15.5–26.0)	<0.001
NRS2002	4.0 (3.0–5.0)	4.0 (3.0–5.0)	5.0 (4.0–6.0)	0.001
Comorbidities (*n* (%))	
Diabetes	53 (21.5%)	40 (20.6%)	13 (24.5%)	0.539
Hypertension	79 (32.0%)	66 (34.0%)	13 (24.5%)	0.189
Coronary heart disease	26 (10.5%)	20 (10.3%)	6 (11.3%)	0.832
Tumor	109 (44.1%)	82 (42.3%)	27 (50.9%)	0.260
Laboratory tests	
WBC (x10^9^/L)	9.61 (6.68–15.14)	9.36 (6.58–15.03)	10.35 (6.83–15.28)	0.639
Lymphocyte (x10^9^/L)	0.62 (0.38–1.01)	0.66 (0.40–1.02)	0.46 (0.34–0.90)	0.071
Neutrophil (x10^9^/L)	8.22 (5.54–13.24)	8.09 (5.57–13.01)	9.11 (5.36–14.05)	0.738
Monocyte (x10^9^/L)	0.47 (0.27–0.75)	0.48 (0.28–0.75)	0.41 (0.20–0.73)	0.274
PLT (× 10^9^/L)	156.0 (96.0–216.0)	163.50 (106.5–219.3)	107.0 (61.5–184.0)	0.001
CRP (mg/L)	41.27 (6.98–137.00)	32.97 (6.73–137.37)	73.14 (9.27–139.84)	0.193
Total protein (g/L)	51.80 (46.10–57.40)	51.75 (46.08–57.25)	51.80 (44.60–59.70)	0.565
Albumin (g/L)	29.07 ± 6.10	29.08 ± 6.12	29.00 ± 6.09	0.932
Prealbumin (mg/L)	101.0 (74.0–142.0)	107.0 (81.5–148.3)	78.0 (55.5–119.5)	<0.001
ALT (U/L)	20.00 (13.00–33.00)	20.00 (13.00–33.25)	18.00 (13.00–35.00)	0.662
AST (U/L)	30.00 (20.00–50.00)	29.00 (19.00–46.25)	36.00 (22.00–78.50)	0.023
Total bilirubin (μmol/L)	13.60 (8.50–22.60)	12.70 (8.28–21.63)	17.60 (9.55–38.45)	0.011
Urea (mmol/L)	8.20 (5.60–13.70)	7.89 (5.30–13.61)	10.00 (6.60–14.35)	0.398
Creatinine (μmol/L)	102.00 (70.00–188.00)	97.50 (69.75–189.25)	112.00 (73.00–199.50)	0.558
Urea-to-creatinine ratio	74.27 (54.55–98.63)	74.14 (54.48–98.37)	74.27 (53.46–102.22)	0.738
eGFR (ml/min/1.73 m^2^)	52.17 (30.87–79.72)	53.69 (31.59–79.16)	44.10 (29.06–82.30)	0.469

BMI, body mass index; SOFA, sequential organ failure assessment; APACHE II, acute physiology and chronic health evaluation II; NRS2002, nutritional risk screening 2002; WBC, white blood cell; PLT, platelet; CRP, C reaction protein; ALT, alanine aminotransferase; AST, aspartate transaminase; eGFR, estimated glomerular filtration rate.

### 3.2 Comparison of ultrasound-derived muscle indices between the survivor and non- survivor group

**Figure 1:**
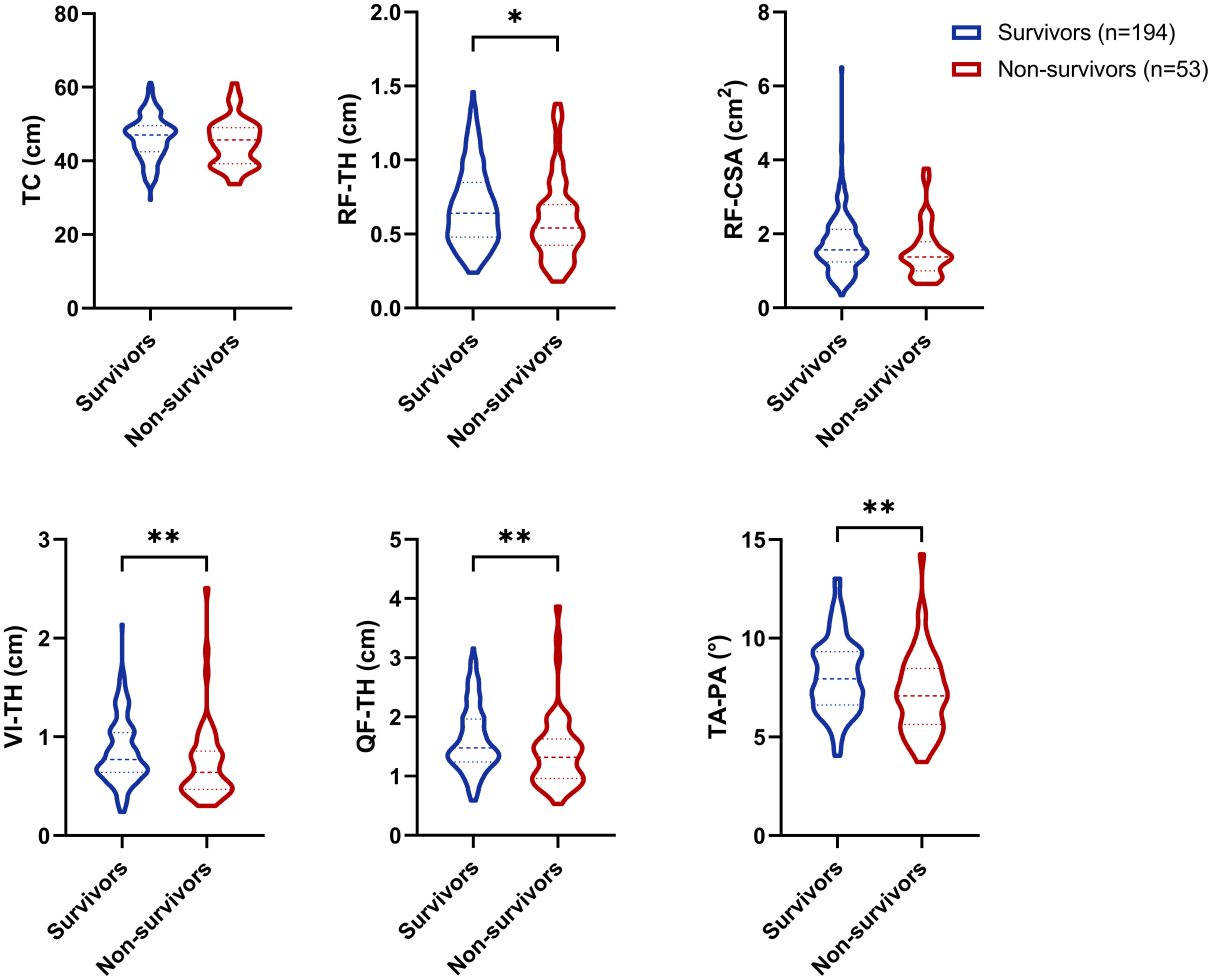
Comparison of different muscle ultrasound metrics between survivor and non-survivor groups *p < 0.05; **p < 0.01. TC, thigh circumference; RF-TH, rectus femoris thickness; VI-TH, vastus intermedius thickness; QF-TH, quadriceps femoris thickness; RF-CSA, rectus femoris cross-sectional area; TA-PA, tibialis anterior pennation angle.

Comparison to the survivor group, the non-survivor group exhibited significantly lower values for RF-TH (0.54 [0.43–0.70] vs. 0.64 [0.48–0.85] cm; *P* = 0.022), VI-TH (0.64 [0.47–0.86] vs. 0.77 [0.64–1.04] cm; *P* = 0.002), QF-TH (1.32 [0.96–1.63] vs. 1.48 [1.24–1.97] cm; *P* = 0.005), and TA-PA (7.08 [5.63–8.48] vs. 7.94 [6.62–9.32]; *P* = 0.002). Additionally, the non-survivor group appeared to have a lower RF-CSA (1.38 [1.00–1.79] vs. 1.57 [1.24–2.12] cm^2^; *P* = 0.065), although this difference did not reach statistical significance (Figure 1).

### 3.3 Relationship between ultrasound muscle indices and 60-day mortality

Univariable model analysis showed that SOFA score(OR = 1.199, 95%CI: 1.107–1.299, *P* < 0.001), APACHE II score (OR = 1.140, 95%CI: 1.082–1.201, *P* < 0.001), NRS2002 score (OR = 1.518, 95%CI: 1.188–1.940, *P* = 0.001), PLT (OR = 0.994, 95%CI: 0.990–0.998, *P* = 0.003), prealbumin (OR = 0.989, 95%CI: 0.983–0.996, *P* = 0.001), RF-TH (OR = 0.269, 95%CI: 0.077–0.944, *P* = 0.040), VI-TH (OR = 0.319, 95%CI: 0.113–0.904, *P* = 0.032), QF-TH (OR = 0.494, 95%CI: 0.268–0.910, *P* = 0.024), TA-PA (OR = 0.777, 95%CI: 0.652–0.926, *P* = 0.005) and was significantly correlated with 60-day mortality (Supplementary Table S3). RF-TH, VI-TH and QF-TH exhibited multicollinearity with VIF>10, therefore, among muscle ultrasound indices, only TA-PA was retained in the multivariable model. Multivariable logistic regression was performed in both complete-case data and the five imputed datasets. The same predictor variables were retained in the final model across all analyses. In the complete-case analysis, TA-PA (OR = 0.806, 95%CI: 0.659–0.985, *P* = 0.035), prealbumin level (OR = 0.992, 95%CI: 0.985–0.999, *P* = 0.029) and APACHE II score (OR = 1.117, 95%CI: 1.057–1.180, *P* < 0.001) were identified as independent predictors of 60-day mortality (Supplementary Table S4). The same three variables were statistically significant in all five imputed datasets (Supplementary Table S5). In the merged results, TA-PA (OR = 0.809, 95%CI: 0.665–0.984, *P* = 0.034), prealbumin level (OR = 0.992, 95%CI: 0.985–0.999, *P* = 0.020) and APACHE II score (OR = 1.126, 95%CI: 1.066–1.189, *P* < 0.001) were still identified as independent predictors (Supplementary Table S4).

Prealbumin is a negative acute-phase reactant, and its circulating level is jointly affected by inflammation and nutritional status. To account for this dual influence, CRP was included as a covariate for further analysis. Previous studies have demonstrated that prealbumin levels below 100 mg/L are significantly associated with poor patient prognosis (30, 31), whereas CRP levels exceeding 50 mg/L indicate a state of hyper-inflammation, often serving as a critical threshold for initiating antimicrobial therapy in various infectious diseases (32, 33). CRP levels were dichotomized accordingly, with participants having CRP levels <50 mg/L serving as the reference group. Prealbumin levels were dichotomized at the cutoff of 100 mg/L, with participants having prealbumin levels ≥100 mg/L serving as the reference group. In this model, a prealbumin level <100 mg/L (OR = 3.921, 95%CI: 1.405–10.944, *P* = 0.009), TA-PA (OR = 0.828, 95%CI: 0.687–0.997, *P* = 0.046), and the APACHE II score (OR = 1.135, 95%CI: 1.075–1.199, *P* < 0.001) remained independent predictors of 60-day mortality in pooled imputed analysis (Table 2). Neither CRP nor the interaction term between prealbumin and CRP attained statistical significance (Table 2). In addition, we performed a stratified sensitivity analysis based on different centers. While no statistically significant association was observed between TA-PA and 60-day mortality in one of the centers, the trends across both centers were consistent (Supplementary Table S6).

**Table 2:** Multivariable logistic regression of 60-day mortality.

Variables	Complete-case analysis (***n*** = 227)			Pooled imputed analysis (***n*** = 247)		
	β	OR (95%CI)	*P*	β	OR (95%CI)	*P*
Prealbumin						
≥100 mg/L (reference)	–	–	–	–	–	–
<100 mg/L	1.352	3.865 (1.377–10.852)	0.010	1.366	3.921 (1.405–10.944)	0.009
CRP						
<50 mg/L (reference)	–	–	–	–	–	–
≥50 mg/L	0.337	1.401 (0.459–4.276)	0.554	0.421	1.523 (0.500–4.634)	0.459
**Prealbumin** ^*^ **CRP**	−0.809	0.445 (0.108–1.843)	0.264	−0.800	0.449 (0.109–1.852)	0.268
**TA-PA**	−0.200	0.819 (0.675–0.992)	0.042	−0.189	0.828 (0.687–0.997)	0.046
**APACHE II**	0.120	1.128 (1.068–1.192)	<0.001	0.127	1.135 (1.075–1.199)	<0.001

CRP, C-reactive protein; TA-PA, tibialis anterior pennation angle; APACHE II, acute physiology and chronic health evaluation II; OR, odds ratio; CI, confidence interval.

### 3.4 Nomogram construction

**Figure 2:**
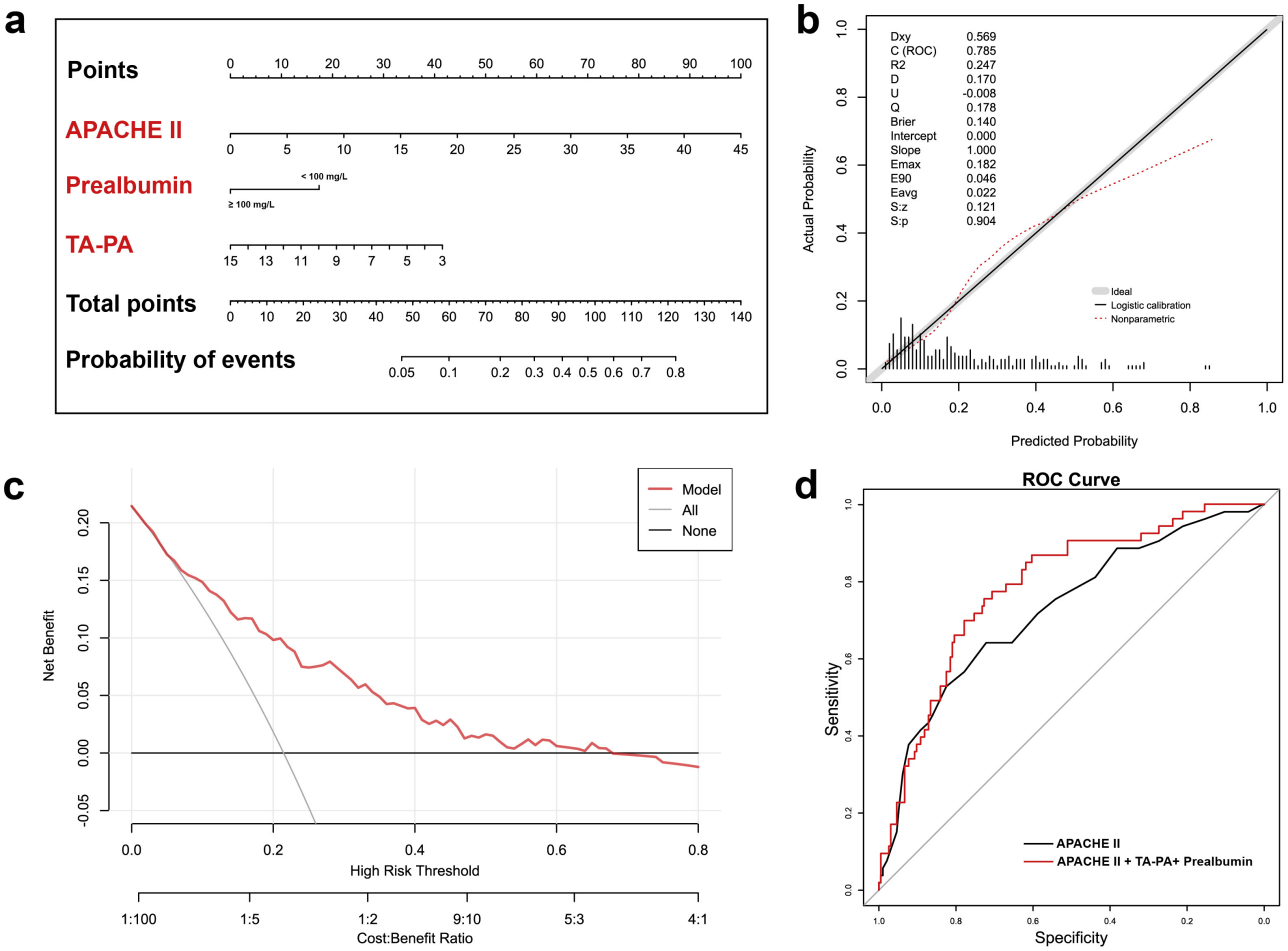
Nomogram of predicting 60-day mortality of the critical ill patients **(a)** Nomogram comprised of APACHE II score, prealbumin and TA-PA. **(b)** Calibration curve for the nomogram. **(c)** Decision curve analysis. **(d)** Comparison of two ROC for 60-day death. APACHE II, acute physiology and chronic health evaluation II; TA-PA, tibialis anterior pennation angle; Dxy, rank correlation coefficient; C-index, area under the ROC curve; R^2^, Nagelkerke-Cox-Snell-Maddala-Magee R-squared index; D, discrimination index; U, unreliability index; Q, quality index; Emax, the maximum absolute difference between the predicted value and the actual value; Eavg, the average difference between the predicted value and the actual value; S:z/S:p, the *Z*-value and *P*-value of Spiegelhalter Z-test.

According to the multivariable regression analysis, TA-PA, prealbumin level and APACHEII score were integrated to develop the nomogram to predict 60-day mortality (Figure 2a). The C-index for the nomogram was 0.785 (95%CI: 0.728–0.834). The nomogram assigns points to each parameter based on a point scale. The sum of these points provides a total score, which can be used to estimate the risk of 60-day mortality. Calibration curve demonstrated that fitted curve conformed with reference line (S:p = 0.904 > 0.05) indicated that the model had good performance in calibration (Figure 2b). DCA curves indicated the superior efficacy of the model in predicting 60-day mortality (Figure 2c). The corrected C-index of the nomogram model obtained from bootstrap resampling was 0.785 (95%CI: 0.719–0.852), indicating good internal validation. Based on the principle of maximizing net returns in decision curve analysis and Youden index of ROC, 0.25 may be adopted as the high-risk cutoff which corresponds to a total point of nomogram around 80. In addition, APACHE II scores integrated with TA-PA and prealbumin had higher AUC to predict 60-day death (0.785 (95%CI: 0.728–0.834) vs. 0.726 (95%CI: 0.666–0.781), *P* = 0.023) than APACHE II scores alone (Figure 2d).

### 3.5 Comparison of clinical characteristics between the low TA-PA and high TA-PA group

Base on the median value of TA-PA, participants were categorized into two groups: those with TA-PA below 7.70° were classified as the low TA-PA group, while those with values equal to or above 7.70° were classified as the high TA-PA group. The low TA-PA group exhibited a lower proportion of males (59.8% vs. 78.4%; *P* = 0.002), a lower BMI (21.50 ±3.75 vs. 23.61 ± 3.09Kg/m^2^; *P* < 0.001), and higher scores on the both APACHEII (17.0 [13.0–22.0] vs. 15.0 [11.0–19.0]; *P* = 0.049) and NRS2002 (5.0 [4.0–6.0] vs. 4.0 [3.0–5.0]; *P* < 0.001). Additionally, the prevalence of sepsis diagnosis was higher in the low TA-PA group (53.3% vs. 37.6%; *P* = 0.013). Furthermore, the TA-PA group exhibited higher CRP levels (62.30 [13.03–155.22] mg/L vs. 21.96 [4.73–122.65] mg/L; *P* = 0.028) and urea-to-creatinine ratio (83.63 [53.95–111.26] vs. 67.76 [54.70–91.30] cm; *P* = 0.012). (Table 3).

**Table 3:** Comparison of clinical characteristics between the low and high TA-PA groups.

Items	Low TA-PA group (TA-PA <7.70, *n* = 122)	High TA-PA group (TA-PA≥7.70, *n* = 125)	*P*
Age (years)	61.50 (51.00–72.00)	60.00 (51.00–72.00)	0.911
Gender (male, *n* (%))	73 (59.8%)	98 (78.4%)	0.002
BMI (kg/m^2^)	21.50 ± 3.75	23.61 ± 3.09	<0.001
SOFA	8.0 (5.0–12.0)	7.0 (5.0–11.0)	0.331
APACHE II	17.0 (13.0–22.0)	15.0 (11.0–19.0)	0.049
NRS2002	5.0 (4.0–6.0)	4.0 (3.0–5.0)	<0.001
Comorbidities (*n* (%))			
Diabetes	26 (21.3%)	27 (21.6%)	0.956
Hypertension	40 (32.8%)	39 (31.2%)	0.789
Coronary heart disease	8 (6.6%)	18 (14.4%)	0.045
Tumor	55 (45.1%)	54 (43.2%)	0.767
Diagnosis (*n* (%))			
Acute kidney injury	29 (23.8%)	21 (16.8%)	0.173
ARDS	5 (4.1%)	12 (9.6%)	0.130
Sepsis	65 (53.3%)	47 (37.6%)	0.013
Laboratory tests			
WBC (× 10^9^/L)	8.81 (6.14–15.08)	10.32 (7.08–15.24)	0.155
Lymphocyte (× 10^9^/L)	0.61 (0.34–1.00)	0.63 (0.42–1.02)	0.454
Neutrophil (× 10^9^/L)	7.51 (5.30–13.00)	8.83 (5.81–13.89)	0.084
Monocyte (× 10^9^/L)	0.49 (0.28–0.75)	0.44 (0.26–0.75)	0.882
PLT (× 10^9^/L)	148.50 (96.75–220.25)	159.00 (94.00–202.50)	0.996
CRP (mg/L)	62.30 (13.03–155.22)	21.96 (4.73–122.65)	0.028
Total protein (g/L)	52.75 (46.98–57.40)	51.10 (45.05–57.40)	0.245
Albumin (g/L)	29.41 ± 5.62	28.73 ± 6.54	0.386
Prealbumin (mg/L)	101.00 (74.50–136.00)	100.50 (73.50–167.50)	0.629
ALT (U/L)	18.50 (13.00–33.00)	22.00 (13.00–34.50)	0.458
AST (U/L)	30.50 (21.00–54.50)	29.00 (19.00–47.50)	0.483
Urea (mmol/L)	9.40 (5.68–16.15)	7.60 (5.25–12.90)	0.117
Creatinine (μmol/L)	97.00 (64.25–225.75)	103.10 (74.85–167.00)	0.495
Urea-to-creatinine ratio	83.63 (53.95–111.26)	67.76 (54.70–91.30)	0.012
eGFR (mL/min/1.73 m^2^)	44.23 (25.36–79.98)	53.82 (35.79–80.97)	0.147
Clinical outcomes			
Mechanical ventilation	–	–	–
Yes (*n* (%))	95 (77.9%)	95 (76.0%)	0.729
Duration (days)	4.0 (2.0–8.0)	4.0 (2.0–8.0)	0.857
Hospital stays (days)	15.5 (10.0–29.3)	17.0 (10.5–27.0)	0.623
ICU stays (days)	5.0 (3.0–9.0)	5.0 (3.0–8.0)	0.706
60-day mortality (*n* (%))	33 (27.0%)	20 (17.6%)	0.035

BMI, body mass index; SOFA, sequential organ failure assessment; APACHE II, acute physiology and chronic health evaluation II; NRS2002, nutritional risk screening 2002; ARDS, acute respiratory distress syndrome; WBC, white blood cell; PLT, platelet; CRP, C-reactive protein; ALT, alanine aminotransferase; AST, aspartate transaminase; eGFR, estimated glomerular filtration rate.

## 4 Discussion

In this study, a dural-center, prospective observational study was conducted to investigate the relationship between ultrasound-derived muscle indices at ICU admission and 60-day mortality. The results indicated that TA-PA was an independent predictor factor of 60-day mortality. Furthermore, a nomogram model encompassing TA-PA demonstrated a good predictive performance for 60-day mortality. These findings offer a novel insight to facilitate the early risk stratification of 60-day mortality in critically ill patients.

Skeletal muscle function is based on muscle quantity and quality. PA is one of the qualitative metrics, referring to the angle at which muscle fibers insert into the aponeurosis, reflecting muscle strength. Specifically, a larger PA indicates a greater amount of contractile material packed within a given volume, thereby enhancing the muscle's capacity to generate force (34). It is also a geometric parameter intrinsically linked to fascicle length, which is determined by the number of sarcomeres arranged in series. During the early stage of immobilization, the primary adaptive response of skeletal muscle is the rapid loss of sarcomeres in series (35), which leads to immediate fiber shortening and a consequent reduction in PA. Moreover, it serves as an integrated indicator of sarcomere length adaptation, mechanical functional integrity (36), and dynamic responsiveness to systemic metabolic and inflammatory cues (37). While macroscopic morphometric indices such as CSA and thickness, represents the cumulative, downstream consequences of these underlying microstructural and functional adaptations, meaning their changes exhibit a temporal delay (38). Moreover, a significant correlation was observed between the PA and the CSA (39), suggesting that the PA could potentially serve as an informative marker integrating both quantitative and qualitative characteristics of muscles. Previous studies had reported that RF-TA, VI-PA and the PA of gastrocnemius medialis were helpful to identify frailty in older people (40–42). TA-PA is utilized in the rehabilitation assessment of stroke patients (43), but has not yet been investigated in critically ill patients. The TA, which primarily facilitates dorsiflexion of the foot and enable the upward movement of the toes, is integral to maintaining a stable gait, maintaining balance, and stabilizing the ankle joint during standing and walking. Previous studies have shown that the improved TA-PA in patients with subacute stroke led to enhanced ankle dorsiflexion strength and improved motor function (44). It has been confirmed that the improved stand ability of critically ill patients was significantly correlated with decreased odds of readmission to the ICU, and reduced 1-year mortality risk (45, 46). This suggests that the motor function related to TA-PA may be closely related to the prognosis of critically ill patients. In addition, our results found that the low TA-PA group exhibited significantly higher CRP levels and urea-to-creatinine ratio, which suggested that low TA-PA was closely associated with inflammation and catabolic status. Skeletal muscle serves as a critical physiological reserve within the human body. During periods of physiological stress, it swiftly mobilizes energy by catabolizing glycogen to sustain essential organs or immune cells. Concurrently, it releases amino acids through catabolic pathways, which are instrumental in the synthesis of acute-phase proteins, thereby facilitating the body's stress response. Research spanning various clinical conditions, such as liver transplantation, and cirrhosis (47, 48), has consistently shown that individuals with diminished muscle quality experience poorer clinical outcomes and higher mortality rates compared to those with normal muscle quality.

Moreover, the integration of muscle-related indicators into prognostic assessments has gained significant attention across various diseases, providing a more accurate basis for subsequent therapeutic interventions. For instance, temporal muscle thickness, mean corpuscular volume and prognostic nutritional index were employed to construct a prognostic prediction model for glioblastoma, achieving an area under the curve of 0.945 for predicting 6-month survival rates (49). Additionally, a combination of triceps skinfold thickness, calf circumference or grip strength-to-body weight ratio, and indicators of reduced food intake was utilized to predict two-year overall survival in patients with upper gastrointestinal cancer, showing significantly enhanced performance compared to the Patient-generated subjective global assessment (PG-SGA) (50). While it has been suggested that incorporating muscle quality or quantity at ICU admission into prognostic assessments could enhance the evaluation of patient outcomes (20), there is currently a lack of relevant studies in this area. In response, we preliminary developed the novel predictive model that integrates TA-PA, prealbumin levels, and the APACHE II score. The APACHE II score is widely used for assessing disease severity, covering multiple systems such as respiratory, circulatory and neurological systems. By quantifying acute physiological parameters, age and chronic health conditions, it comprehensively reflects the multi-organ functional status and predicts mortality risk. Skeletal muscle serves as a reserve organ and is closely linked to various functions such as immunity, metabolism and movement. The muscle status at ICU admission is closely related to the patient's prior health conditions.

Prealbumin, in addition to being an important nutritional indicator, is also a negative acute-phase protein (51). During the acute phase, prealbumin synthesis was depressed by CRP and other inflammatory mediators (52). In this study, prealbumin remained a significant independent predictor of 60-day mortality even after adjusting for CRP levels and including an interaction term between prealbumin and CRP predictor value. The persistence of this association suggests that the prognostic value of prealbumin is not solely mediated by the acute-phase response, and the adverse prognostic impact of low prealbumin is consistent across different levels of inflammation. The predictive performance of the model integrating TA-PA and prealbumin indicators with the APACHE II score is better than the that of using the APACHE II score alone, improving the predictive ability of 60-day mortality. This provides a new perspective for evaluating the prognosis of critically ill patients. When the body encounters stress, survival-related factors include not only stress-induced damage, but also the individual's baseline physiological state and reserve capacity. Different diseases consume different muscles preferentially, and further validation with larger sample sizes and more muscle indicators from different body regions is still needed.

RF-CSA is most widely studied across different diseases. Umbrello et al. found that low baseline RF-CSA was significantly associated with increased ICU mortality, and serves as an independent risk factor for ICU mortality (18). However, our study did not find a correlation between RF-CSA at ICU admission and 60-day mortality, although non-survivors presented slightly lower RF-CSA values. Several differences were identified between the two studies. Firstly, there is a discrepancy in the timing of ultrasonic testing. Umbrello et al.'s study conducted testing within 48 h of ICU admission, whereas our study involved collecting ultrasound data within 24 h. This variation suggests that TA-PA may exhibit greater sensitivity in prognostic prediction. Secondly, the patient populations differed significantly. Umbrello et al. focused on a European cohort with an average BMI exceeding 25, while our research examined an East Asian cohort with an average BMI below 25. Furthermore, the severity of illness varied between the studies. In the study conducted by Umbrello et al., the cohort exhibited an average SOFA score of six, whereas our patient population demonstrated a higher average SOFA score of eight. This indicates that our study encompassed patients with a lower BMI but experiencing more severe multiple organ dysfunction compared to those in the cohort of Umbrello et al.'s study. Moreover, a study on skeletal muscle quantity and quality loss in patients with colorectal liver metastases reported findings partially consistent with ours (53). It was found that the loss of psoas muscle index (a muscle quantity index) related to the surgery showed a weak statistical trend with the overall survival rate, while the loss of average muscle radiation attenuation of psoas muscle (a muscle quality index) related to the surgery was significantly associated with a higher incidence of postoperative complications. These observed differences may be attributable to variations in disease stage, baseline patient characteristics, and individual susceptibility. Larger-scale, prospective studies are warranted to validate these associations and to identify appropriate muscle indices tailored on the characteristics of different patient populations.

Our research possesses several notable strengths. Firstly, we investigated and validated a novel muscle ultrasound metrics, TA- PA, which has not been previously utilized in critically ill patients. Compared to the commonly used RF, the ultrasound evaluation of TA offers several advantages: its anatomical location on the anterior aspect of the lower leg renders it superficial and easily accessible. During monitoring, only the anterior aspect of the lower leg needs to be exposed, eliminating the need to reposition the patient's hip, which is particularly advantageous for patients with vascular catheters in the groin area. Secondly, we developed a novel prognostic prediction model that incorporates muscle ultrasound indicators, prealbumin level and the APACHE II score. The parameters included in this model are associated with the age, acute physiological status, chronic health conditions, stress response related marker, and reserve capacity of the body. This integration facilitates a more comprehensive and precise evaluation of patient prognosis, providing new insights into the evaluation of critically ill patients.

Some limitations are acknowledged in this study. First, this predictive model had only undergone internal validation. Although internal validation indicated good predictive performance, to further verify its extrapolation, external validation needs to be improved in the future. Second, this study only performed a single ultrasound measurement at ICU admission. Longitudinal measurements would allow assessment of the rate of muscle atrophy and its dynamic relationship with clinical outcomes, which should be explored in future studies. Third, all patients in the study were of Asian descent, with lower BMI and skeletal muscle mass compared to Western populations, so the results should be interpreted with caution. More than two-thirds of our cohort were male which limited generalizability, further investigation is warranted to elucidate sex-specific differences in muscle architecture and their impact on prognosis. Fourth, although this study involved two centers, the sample size remained relatively small. To further validate the predictive effect of TA-PA, a larger sample size and additional centers would be necessary. Fifth, discrepancy in sensitivity analysis may result from small sample size. Future research designs need to take these into account. Lastly, the median value of TA-PA was used to dichotomize patients for exploratory analysis, we acknowledge that this threshold is data-driven and requires external validation. Future studies are warranted to establish and validate clinically meaningful cut-off values for TA-PA in diverse ICU populations.

## 5 Conclusion

This study identified TA-PA is an independent predictor factor of 60-day mortality. By integrating TA-PA with prealbumin levels and the APACHE II score, we developed a nomogram for predicting the 60-day mortality rate, which demonstrated superior predictive performance compared to predictions based solely on the APACHE II score. Early identification of high-risk patients using this nomogram may guide targeted interventions such as early mobilization and closer nutritional monitoring to potentially improve outcomes. These findings enhance the dimensionality of prognostic assessment for critically ill patients and offer potential strategies for early risk stratification of 60-day mortality.

## Data Availability

The raw data supporting the conclusions of this article will be made available by the authors, without undue reservation.
