# Supplementary material for: Tibialis anterior pennation angle at ICU admission and 60-day mortality in critically ill patients: a prospective observational study
Source: Front Med (Lausanne). 2026 Apr 22;13:1815137. doi: 10.3389/fmed.2026.1815137 (PMC13144024; doi:10.3389/fmed.2026.1815137)
Supplement: Supplementary file 1 [file Data_Sheet_1.pdf]

Tibialis anterior pennation angle at ICU admission and 60-day mortality in critically ill patients: a prospective observational study

Supplementary Material

Contents

Figure S1. Schematic diagram of muscle ultrasound measurements 1

Figure S2. Flowchart of participants.. 2

Table S1. Reproducibility of measurements by researchers 3

Table S2. Details of missing data 4

Table S3. Univariable logistic regression of 60-day mortality and multicollinearity test.................... 5

Table S4. Multivariable logistic regression of 60-day mortality 6

Table S5. Multivariable logistic regression in different multiple imputation datasets 7

Table S6. Logistic regression for sensitivity analysis.. 8

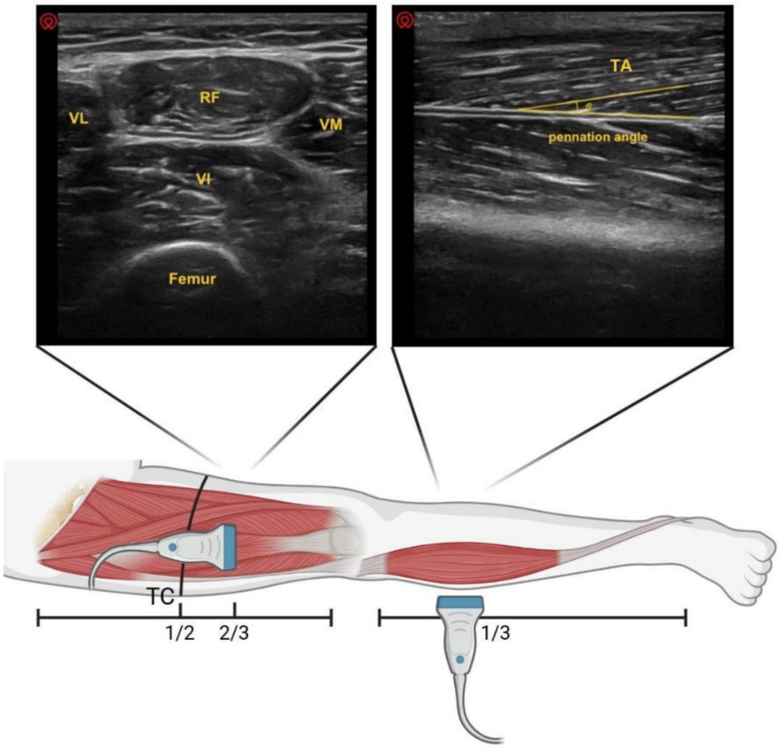

Figure S1. Schematic diagram of muscle ultrasound measurements

RF, rectus femoris. VI, vastus intermedius. VL, vastus lateralis. VM, vastus medialis; TA, tibialis anterior. Created by BioRender (https://www.biorender.com/).

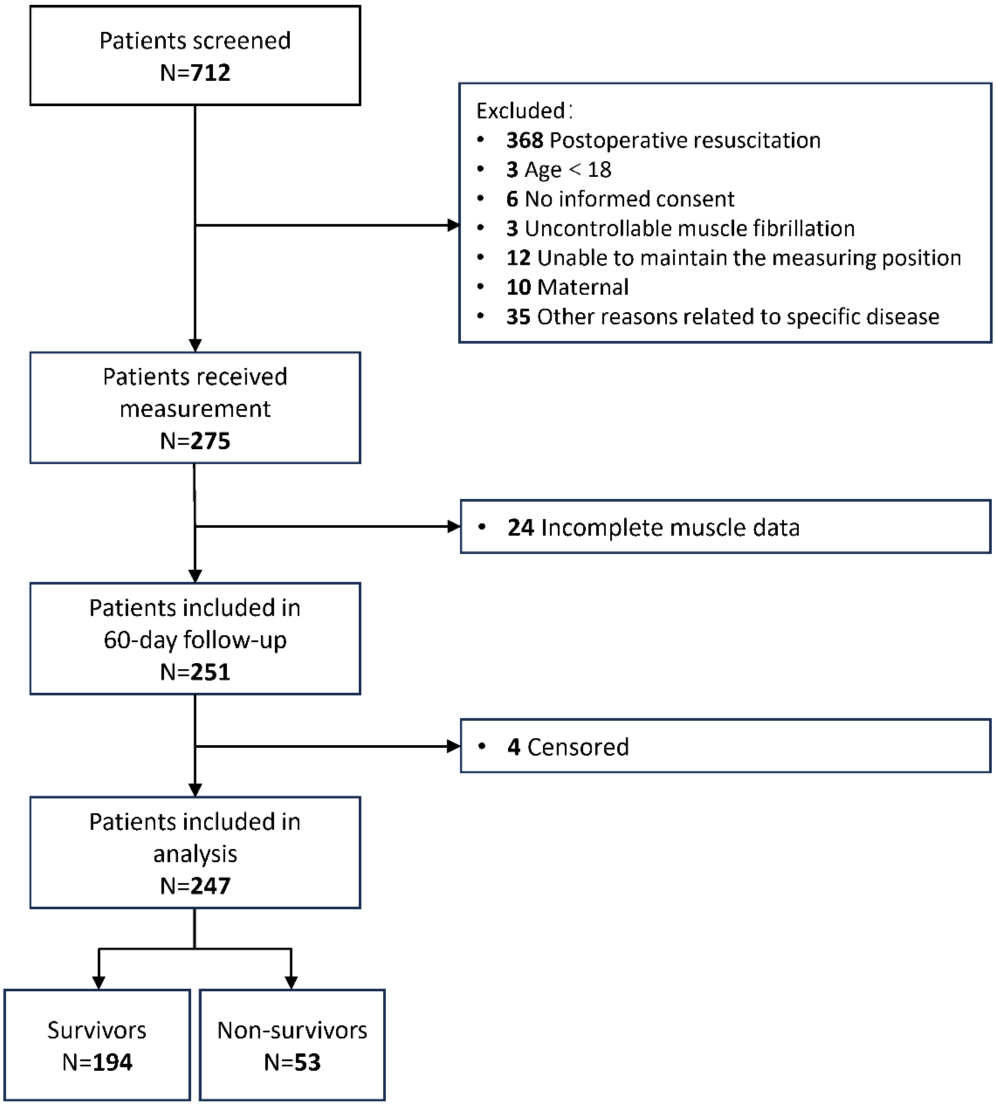

Figure S2. Flowchart of participants

Table S1. Reproducibility of measurements by researchers

Items	Intraclass Correlation Coefficient (95%CI)		
**Intra-observer reliability**	**Researcher 1**	**Researcher 2**	**Researcher 3**
TC	0.998 (0.993-0.999)	0.997 (0.989-0.999)	0.997 (0.986-0.999)
RF-TH	0.994 (0.976-0.999)	0.996 (0.984-0.999)	0.997 (0.988-0.999)
VI-TH	0.993 (0.971-0.998)	0.994 (0.976-0.998)	0.993 (0.973-0.998)
QF-TH	0.995 (0.979-0.999)	0.998 (0.991-0.999)	0.994 (0.976-0.998)
RF-CSA	0.991 (0.277-0.999)	0.989 (0.956-0.997)	0.995 (0.983-0.999)
TA-PA	0.946 (0.783-0.987)	0.946 (0.795-0.986)	0.903 (0.628-0.976)
**Inter-observer reliability**	**Researchers**		
TC	0.988 (0.966-0.997)		
RF-TH	0.991 (0.975-0.998)		
VI-TH	0.986 (0.956-0.996)		
QF-TH	0.989 (0.968-0.997)		
RF-CSA	0.985 (0.957-0.996)		
TA-PA	0.927 (0.809-0.979)		

Abbreviations: CI: confidence interval; TC: thigh circumference. RF-TH, rectus femoris thickness; VI-TH, vastus intermedius thickness. QF-TH, quadriceps femoris thickness; RF- CSA, rectus femoris cross-sectional area; TA-PA, tibialis anterior pennation angle.

Reproducibility of researchers was assessed for 10 patients. For each region, two independent images were acquired and measurements were obtained directly at the bedside employing the integrated measurement scale of the ultrasound device. The consistency test was conducted independently, and the operator was unaware of the results of others and the patient's medical records.

Table S2. Details of missing data

Items	Data type	Missing values (%)
Age (years)	Continuous variable	0
Gender (male, n (%))	Binary variable	0
BMI (kg/m^2)	Continuous variable	0
SOFA	Continuous variable	0
APACHE II	Continuous variable	0
NRS2002	Continuous variable	0
Diagnosis	Categorical variable	0
WBC ($x10^9/L$)	Continuous variable	0
Lymphocyte ($x10^9/L$)	Continuous variable	0
Neutrophil ($x10^9/L$)	Continuous variable	0
Monocyte ($x10^9/L$)	Continuous variable	0
PLT ($x10^9/L$)	Continuous variable	0
CRP (mg/L)	Continuous variable	1 (0.4%)
Total protein (g/L)	Continuous variable	9 (3.6%)
Albumin (g/L)	Continuous variable	9 (3.6%)
Prealbumin (mg/L)	Continuous variable	20 (8.1%)
ALT (U/L)	Continuous variable	0
AST (U/L)	Continuous variable	0
Urea (mmol/L)	Continuous variable	0
Creatinine (µmol/L)	Continuous variable	0
Urea-to-creatinine ratio	Continuous variable	0
eGFR (mL/min/1.73 m^2)	Continuous variable	0
Mechanical ventilation (days)	Continuous variable	0
Hospital stays (days)	Continuous variable	0
ICU stays (days)	Continuous variable	0
TC (cm)	Continuous variable	0
RF-TH (cm)	Continuous variable	0
VI-TH (cm)	Continuous variable	0
QF-TH (cm)	Continuous variable	0
RF-CSA (cm^2)	Continuous variable	0
TA-PA (°)	Continuous variable	0

Abbreviations: BMI, body mass index; SOFA, sequential organ failure assessment; APACHE II, acute physiology and chronic health evaluation II; NRS2002, nutritional risk screening 2002; WBC, white blood cell; PLT, blood platelet; CRP, C reaction protein; ALT, alanine aminotransferase; AST, aspartate transaminase; eGFR, estimated glomerular filtration rate; TC, thigh circumference. RF-TH, rectus femoris thickness; VI-TH, vastus intermedius thickness. QF-TH, quadriceps femoris thickness; RF- CSA, rectus femoris cross-sectional area; TA-PA, tibialis anterior pennation angle.

Table S3. Univariable logistic regression of 60-day mortality and multicollinearity test

Variables	OR (95%CI)	*P*	VIF
Age	1.003 (0.983-1.025)	0.743	1.155
Gender	1.308 (0.662-2.586)	0.439	1.425
BMI	0.978 (0.898-1.065)	0.614	1.318
SOFA	1.199 (1.107-1.299)	< 0.001	1.916
APACHE II	1.140 (1.082-1.201)	< 0.001	1.668
NRS2002	1.518 (1.188-1.940)	0.001	1.792
PLT	0.994 (0.990-0.998)	0.003	1.433
Prealbumin	0.989 (0.983-0.996)	0.001	1.228
AST	1.003 (0.998-1.009)	0.193	1.065
RF-TH	0.269 (0.077-0.944)	0.040	10.263
VI-TH	0.319 (0.113-0.904)	0.032	11.676
QF-TH	0.494 (0.268-0.910)	0.024	31.123
TA-PA	0.777 (0.652-0.926)	0.005	1.390

Abbreviations: BMI, body mass index; SOFA, sequential organ failure assessment; APACHE II, acute physiology and chronic health evaluation II; NRS2002, nutritional risk screening 2002; PLT, platelet; AST, aspartate transaminase; RF-TH, rectus femoris thickness; VI-TH, vastus intermedius thickness; QF-TH, quadriceps femoris thickness; TA-PA, tibialis anterior pennation angle; VIF, variance inflation factor.

Table S4. Multivariable logistic regression of 60-day mortality

	**Complete -case analysis(n=227)**			**Pooled imputed analysis(n=247)**		
**Variables**	**β**	**OR (95%CI)**	***P***	**β**	**OR (95%CI)**	***P***
TA-PA	-0.216	0.806 (0.659-0.985)	0.035	-0.211	0.809 (0.665-0.984)	0.034
PLT	-0.004	0.996 (0.992-1.001)	0.112	-0.004	0.996 (0.992-1.001)	0.103
Prealbumin	-0.008	0.992 (0.985-0.999)	0.029	-0.008	0.992 (0.985-0.999)	0.020
BMI	0.035	1.036 (0.937-1.145)	0.494	0.045	1.047 (0.948-1.156)	0.370
APACHE II	0.110	1.117 (1.057-1.180)	<0.001	0.119	1.126 (1.066-1.189)	<0.001

Abbreviations: TA-PA, tibialis anterior pennation angle; PLT, platelet; BMI, body mass index; APACHE II, acute physiology and chronic health evaluation II; OR, odds ratio; CI, confidence interval.

Table S5. Multivariable logistic regression in different multiple imputation datasets

	Dataset 1			Dataset 2			Dataset 3		
**Variables**	**β**	**OR (95%CI)**	***P***	**β**	**OR (95%CI)**	***p***	**β**	**OR (95%CI)**	***P***
TA-PA	-0.209	0.811(0.667-0.987)	0.036	-0.209	0.812(0.668-0.986)	0.036	-0.217	0.805(0.662-0.979)	0.030
PLT	-0.004	0.996(0.992-1.001)	0.098	-0.003	0.997(0.992-1.001)	0.109	-0.003	0.997(0.992-1.001)	0.113
Prealbumin	-0.009	0.991(0.984-0.998)	0.013	-0.009	0.992(0.985-0.998)	0.017	-0.009	0.991(0.984-0.998)	0.013
BMI	0.045	1.046(0.947-1.155)	0.378	0.045	1.046(0.947-1.155)	0.372	0.044	1.045(0.946-1.154)	0.390
APACHE II	0.118	1.126(1.066-1.189)	< 0.001	0.119	1.127(1.066-1.190)	< 0.001	0.120	1.127(1.067-1.191)	< 0.001

	Dataset 4			Dataset 5		
**Variables**	**β**	**OR (95%CI)**	***P***	**β**	**OR (95%CI)**	***P***
TA-PA	-0.202	0.817(0.672-0.993)	0.042	-0.221	0.802(0.660-0.974)	0.026
PLT	-0.004	0.996(0.992-1.001)	0.093	-0.004	0.996(0.992-1.001)	0.102
Prealbumin	-0.008	0.992(0.985-0.999)	0.024	-0.008	0.992(0.985-0.999)	0.029
BMI	0.048	1.049(0.950-1.159)	0.345	0.046	1.047(0.948-1.156)	0.363
APACHE II	0.119	1.127(1.067-1.190)	< 0.001	0.115	1.122(1.063-1.185)	< 0.001

Abbreviations: TA-PA, tibialis anterior pennation angle; PLT, platelet; BMI, body mass index; APACHE II, acute physiology and chronic health evaluation II.

Table S6. Logistic regression for sensitivity analysis

Stratified by center	Univariable analysis			Multivariable analysis		
	**β**	**OR (95%CI)**	***p***	**β**	**OR (95%CI)**	***p***
**Center 1 (N=48)**						
Age	0.011	1.012 (0.961-1.065)	0.662	--	--	--
Gender	-0.595	0.552 (0.107-2.848)	0.478	--	--	--
BMI	0.004	1.004 (0.775-1.302)	0.973	--	--	--
SOFA	0.145	1.156 (0.920-1.451)	0.213	--	--	--
APACHE II	0.294	1.342 (1.104-1.631)	0.003	0.330	1.390 (1.112-1.738)	0.004
NRS2002	0.491	1.634 (0.891-2.998)	0.113	--	--	--
TA-PA	-0.073	0.930 (0.612-1.412)	0.733	-0.083	0.921 (0.595-1.424)	0.710
Prealbumin	-0.006	0.994 (0.983-1.005)	0.278	-0.008	0.992 (0.978-1.006)	0.254
**Center 2 (N=199)**						
Age	-0.001	0.999 (0.976-1.023)	0.940	--	--	--
Gender	0.435	1.545 (0.724-3.293)	0.260	--	--	--
BMI	-0.020	0.980 (0.896-1.073)	0.668	--	--	--
SOFA	0.184	1.202 (1.101-1.311)	< 0.001	--	--	--
APACHE II	0.109	1.115 (1.057-1.177)	< 0.001	0.101	1.107 (1.047-1.170)	< 0.001
NRS2002	0.391	1.479 (1.113-1.965)	0.007	--	--	--
TA-PA	-0.280	0.756 (0.622-0.919)	0.005	-0.218	0.804 (0.656-0.985)	0.036
Prealbumin	-0.013	0.987 (0.980-0.995)	0.002	-0.012	0.989 (0.980-0.997)	0.009

Abbreviations: BMI, body mass index; SOFA, sequential organ failure assessment; APACHE II, acute physiology and chronic health evaluation II; NRS2002, nutritional risk screening 2002; TA-PA, tibialis anterior pennation angle
